# Revisiting caregiver satisfaction with children’s mental health services in the United States

**DOI:** 10.1186/s13033-021-00493-9

**Published:** 2021-08-28

**Authors:** Lauren F. Seibel, Robin Peth-Pierce, Kimberly E. Hoagwood

**Affiliations:** 1 Department of Child and Adolescent Psychiatry, New York University Grossman School of Medicine, 1 Park Ave, 7th Floor, New York, NY 10016 USA; 2 Public Health Communications Consulting, LLC, 16678 State Rd., North Royalton, OH 44133 USA

**Keywords:** Parent satisfaction, Caregiver satisfaction, Child and adolescent mental health services, Community mental health services, Measurement of caregiver satisfaction, Consumer satisfaction

## Abstract

Nearly four decades ago, Unclaimed Children documented the gaps in the United States between mental health programs and caregivers’ perspectives about those services for their children. This absence of attention to parent or caregiver perspectives, including their satisfaction with these services, was a key finding of the report, which detailed system failure in caring for youth with mental health needs. Since then, the focus on caregiver satisfaction with children’s mental health services has been largely overlooked in research, and when examined has been mostly included as an indicator of the feasibility of program implementation. In striking contrast, overall healthcare system reforms have highlighted the importance of improving consumer’s direct experience of care. However, caregiver satisfaction remains largely disconnected to these overall health system reforms, even as reforms focus increasingly on value-based, coordinated and integrated care. In this paper, we review literature from 2010 to 2020, revisit the measurement of caregiver satisfaction, identify how and when it is being measured, and delineate a research agenda to both realign it with health system improvements, refine its focus on expectancies and appropriateness, and root it more firmly in the principles of user experience (UX) and human-centered design (HCD).

## Background

Nearly two decades have passed since the Institute for Healthcare Improvement (IHI) set the bar for improving health care services in the U.S., outlining the trifecta of factors needed to meet and exceed that bar: improving patient experience of care, improving the *health* of populations, and reducing per capita costs of health care, known as the Triple Aim [10]. Technological innovations (e.g. the use of big data in population-level health monitoring and measurement) and legislative mandates (e.g. Patient Protection and Affordable Care Act or ACA) have driven success in meeting some facets of these three broad and bold goals [61], but simultaneous achievement of all three aims remains elusive, especially for some populations [88]. This Triple Aim framework was especially noteworthy because of its direct focus on the *patient experience of care* (which includes quality of and satisfaction with care), which expanded on earlier Institute of Medicine efforts that cited patient-centeredness as one of six ways to bridge the quality chasm [46].

This focus on patient experience of care propelled consumers, the people receiving services, to the forefront, identifying them as the “true north” that should guide the delivery of healthcare. The emergence of this framework was also a turning point in thinking about the quality of services; to achieve quality, providers and health systems now had to pay attention to consumer preferences for services. To capture and standardize consumers’ preferences for services, the Agency for Healthcare Research and Quality (AHRQ) created a survey, called the Consumer Assessment of Healthcare Providers and Systems surveys, or CAHPS, in 1995 [6]. The CAHPS survey has been shown to be reliable and valid, and research has shown that consumers positively rating their experiences on CAHPS surveys is linked to improvements in clinical outcomes and adherence to outcomes [6]. The passage of the ACA mandated that private and public systems use this CAHPS data as a metric in various healthcare settings, such as ambulatory care, hospital care, and adult behavioral health care—but not caregiver satisfaction with children’s mental health (MH) services, although AHRQ piloted a pediatric version of CAHPS nearly two decades ago [4, 13, 45]. During the two decades since, caregivers’ experience with their child’s mental health care has largely been ignored, though decades-old reports have documented the need to better serve these families and children.

Four decades ago, Unclaimed Children [51] detailed the challenges faced by families in receiving MH services for their children. In response, in the early 1990s, SAMSHA implemented the System of Care (SOC) to better attend to the needs of families of youth with severe emotional disturbance by coordinating or ‘wrapping’ services around families, tailored to their needs and relying in part on natural community supports [15, 79, 80]. In 2008, even with significant SOC expansions and funding, Unclaimed Children, Revisited (2008) documented persistent gaps in state policies and programs that still failed to promote family-driven care and integrate the perspective of families into that care [29, 30]. Up until 2008, significant emphasis in children’s MH services research focused on intervention development and outcomes [14, 63], and more recently, on barriers to care, both structural and perceived [17, 66], with relatively little attention to caregiver satisfaction with care or their preferences for such care [48].

Today, the caregiver satisfaction construct remains less well-studied, as described herein, even though the low rates of engagement in children’s MH care services (80% of children in the U.S. who need this care never access it; [12]), especially among impoverished youth and families with complex social needs, makes understanding the care experience through the lens of the caregiver critical. The extant literature tells us that parents are, importantly, the gatekeepers to their children’s mental health care [85], and studies in youth mental health care document that matching services to caregiver preferences can reduce treatment dropout [8]. In comparison to adult healthcare, where a more significant research base links a patient’s subjective experience of care to various outcomes, including clinical adherence [5, 31, 49], decreased readmissions [18, 74], and reduced rates of mortality [39], research on caregiver satisfaction with their child’s MH services is limited.

Healthcare reforms are increasingly funding peer support services to improve patient outcomes. Both public and private healthcare reforms are highlighting the role of children and families as “consumers” in improving the quality of services, and, including family support services delivered by family peer advocates (FPA). Interest and support for these FPA-delivered services has grown—fueled largely by family advocacy groups—and now family support services, provided by credentialed family peer advocates and required in system-of-care (SOC)-funded services, are Medicaid-billable in 32 states [72], a number that has doubled since 2012 [22].

Public-policy-focused research has called for a move from a largely biomedical system (e.g. focused on individual providers) to an approach that integrates caregiver perspectives throughout the continuum of care, as reflected in the Health Transformation Framework [42]. Another example of policy research to integrate caregiver perspectives into the design of children’s MH services is the Center for Medicare and Medicaid Innovation (CMMI) $126 million investment in a seven-state demonstration of the Integrated Care for Kids (InCK) model that “puts children and families first and at the center of coordinated care across child programs” [24]. Some *private sector* health systems are also placing caregivers at the forefront of general health care delivery, linking parents of children with complex health care needs to parent navigators (e.g. National Children’s Hospital), and testing parent navigator service delivery models to more directly attend to their experience of care and improve outcomes [60]. However, few research studies are focused on the direct collection of caregiver experience of their children’s mental healthcare. While consumer satisfaction data are commonly collected in private managed behavioral healthcare and used to assess program quality [1, 36], the types of measures used, how they are used, and the outcomes from their use are not known. This is largely because these satisfaction surveys are conducted within private health systems and not published.

### Theoretical foundations of parent satisfaction with their child’s mental health services

Parent satisfaction has received limited research attention. Two studies [36, 73] attempt to examine theoretical components of caregiver satisfaction with their child’s mental healthcare. Garland et al. [37] explored the determinants of both youth and parent satisfaction with services, which fell into three categories (service entry characteristics, treatment therapist characteristics, and clinical outcomes) but found very few significant effects of these factors. Shafer & Temple [73] describe the development of the Youth Services Survey for families, and the five domains that comprise parent satisfaction with services (i.e., general satisfaction, outcomes, access, cultural sensitivity and participation and access), and document the YSS-F as a reliable and valid measure. The YSS-F (and YSS) are used by state departments of mental health, though no comprehensive analysis of this dataset appears to have been conducted, and more importantly, easily-accessible, time-trend data is not publicly available, to date.

In this paper, we review the literature on caregiver satisfaction with children’s mental health services in the United States. Because mental healthcare provision is so different between countries and cultures, and evaluations of what it means to be “satisfied” with services varies greatly between countries due to different social and cultural contexts, the authors chose to keep this review focused on the services provided to children in the United States. We examine the differences in the measurement of caregiver satisfaction in a trio of settings: in *publicly delivered* mental health systems, in *privately delivered* (e.g. commercial insurance) programs, and in *research studies*, which mostly focus on improving the dissemination and implementation (D&I) of interventions for children. For publicly delivered care (e.g. Substance Abuse and Mental Health Services Administration [SAMHSA] community MH block grant funded care), caregiver satisfaction with services is usually assessed with the Youth Services Survey for Families (YSS-F) [73]. In the papers reviewed, caregiver satisfaction in privately delivered care is most commonly measured using the long-standing Client Services Questionnaire (CSQ), developed by Larsen et al. [55]. However, research studies, particularly those examining D&I science, have largely developed their own ad-hoc scales of caregiver satisfaction.

To clarify the boundaries of this paper, we note that the concept of caregiver satisfaction the authors chose to examine is different from the definition of caregiver engagement (e.g., participation in services) and is also distinct from the notion of the caregiver experiences of care, which assesses their direct *interaction* with the providers. Rather, for this review, the authors looked into how satisfied parents were (afterward) with their child’s care, or how well it met their needs. Occasionally, the concept of satisfaction does overlap with similar constructs such as caregiver expectations or perceptions of care. However, for the purposes of this review, studies were only included which specifically used the word “satisfaction”, either in the title or description of the measure. We conclude the paper outlining a research agenda to focus on caregiver satisfaction with children’s MH services that can advance quality improvement. Because healthcare service reforms are increasingly targeting value, accountability, and outcomes [23, 62], caregiver experiences of care, particularly their satisfaction, are likely to become a more potent driver of quality improvement system changes in both the private and public MH systems.

## Methods

An initial literature search on caregiver satisfaction with children’s mental health services revealed no exact search terms that accurately captured the construct. This may be because there is no Medical Subject Heading (MEsH) for “parent satisfaction” or “caregiver satisfaction”; rather the closest term is “*patient* satisfaction”. MEsH terms are official words or phrases used for indexing articles in PubMed. Because “parent satisfaction” or “caregiver satisfaction” do not exist as MEsH terms, articles on this topic are not grouped or indexed together, and research on this topic is not easy to identify. Thus, obtaining literature on what caregivers think about the mental health services that their child receives was not a clear-cut process. Using an iterative process, the authors employed broader search terms used in PubMed: “((child) OR (adolescent) OR (youth)) AND ((parent) OR (family) OR (caregiver)) AND (satisfaction) AND ((mental health) OR ("behavioral health")) AND ((intervention) OR (treatment) OR (services)) AND United States[pl]”, with the date filter set to include papers published in the last decade (2010–2020). This search yielded 219 results, and after reviewing abstracts and occasionally full texts, the papers were included if they had a rigorous design (a randomized controlled trial or a prospective longitudinal design), measured caregiver satisfaction with their child’s mental health services, and were conducted in a domestic (United States) population. Only 17 papers met these criteria. The remaining 202 papers were excluded for various reasons, including: studying an international population; measuring an adult population’s satisfaction with their own services; design issues (e.g. case studies or focus group interviews); assessing caregiver satisfaction with issues not related to their child’s care (ex: life satisfaction, parenting satisfaction).

Ten additional papers were identified through subsequent searches. Two papers were added from a librarian search (using terms in PubMed: “(("Parents' Perceptions") OR ("parent satisfaction")) OR ("parents' satisfaction")) OR ("parent's perception")) OR ("parental satisfaction")) OR ("carer satisfaction")) OR (consumer satisfaction OR "caregiver satisfaction")) AND ((((Child) OR (preschool children)) OR (Young Adult)) OR (adolescent))) AND ((((("Surveys and Questionnaires"[Mesh]) OR ( "Surveys and Questionnaires/epidemiology"[Mesh] OR "Surveys and Questionnaires/methods"[Mesh])) OR (Surveys)) OR (Respondent)) OR (tools))) AND (((("Mental Health Services"[Majr]) OR ( "Mental Health Services/methods"[Mesh] OR "Mental Health Services/organization and administration"[Mesh])) OR (mental health care services)) OR (Child and Adolescent Mental Health Services))”. The other eight papers were added through snow-balling strategies, checking for other relevant articles in the citations of the papers we found in our original search, and via reverse snowballing, citation tracing of more recent articles that had cited the articles we had already included. These additional searches yielded a final total of 27 papers, which were then reviewed by all authors. The authors discussed the articles and what were the most important components to present for this review.

## Results

Table 1 categorizes the 27 papers under five broad headings: Conceptualization, Measure, Use, Results, and Service Setting. The “use” column was further sub-divided according to how the construct of satisfaction is used in the research: either as (a) a feasibility component for new intervention; (b) as an outcome for established interventions; or (c) in relationship to clinical, population, or service variables.

**Table 1:** Research articles published 2010-2020 on caregiver satisfaction with youth mental health services in the United States

First Author	Pub. Year	Conceptualization	Measure	Use	Results	Service Setting	Notes
Acri	2016	Defined as viewing the process and outcomes associated with treatment favorably; parents’ opinions about helpfulness of groups, the importance of therapy for families, and family improvements as a result of treatment	Metropolitan Area Child Study process measures program satisfaction subscale	Satisfaction in relationship to clinical, population, or service variables	Satisfaction with treatment was predictive of reductions in problematic child behaviors and parent stress independently; no difference between treatment groups	Outpatient clinic	
Beltran	2016	Indicator of engagement in mental health services	Study-specific researcher-developed measure	Satisfaction as feasibility component for new intervention	Satisfaction ratings were high for parents & children	Outpatient clinic	Also measured child satisfaction
Bonach	2010	Examining how performance of different multidisciplinary agencies vary in the eyes of the consumer, how they shape consumer's experience; find ways to improve and refine service delivery	Study-specific researcher-developed measure	Satisfaction in relationship to clinical, population, or service variables	Caregivers reported fairly high satisfaction with Children's Advocacy Center (CAC). Separated CAC functions, showing that satisfaction with services delivered by the CAC (information and logistical coordination, responsiveness and provision of comfort for child victims and non-offending caregivers, and staff courteousness and helpfulness) was important in predicting overall CAC satisfaction	Children's advocacy center	
Burt	2014	Caregiver satisfaction with the discussion of behavioral health topics with the provider	Promoting Healthy Development Survey- Modified	Satisfaction as outcome for established intervention	Intervention group (integrating behavioral health provider into well-child visit) parent satisfaction was not higher than control (standard well-child visit); overall satisfaction was highly related to perceived helpfulness	Primary care	
Cama	2020	Parents’ satisfaction with the role of the primary care physician (PCP) in the treatment of their child’s mental health problems after their PCP consulted Massachusetts Child Psychiatry Access Project (MCPAP)	Study-specific researcher-developed measure	Satisfaction as outcome for established intervention	Parents expressed high rates of satisfaction; positive patient-doctor relationship related to higher satisfaction	Primary care	
Chavira	2014	Parent level of satisfaction with the intervention that their child received	Parent Consumer Satisfaction Scale	Satisfaction as outcome for established intervention	Parent satisfaction at post-treatment and 3-month follow-up was high for both groups, no significant difference between treatment groups	Cross-Setting: outpatient (control) and telehealth (intervention)	
Coker	2019	Examined satisfaction with the referral process and the care received	2 adapted items from the Consumer Assessment of Healthcare Providers and Systems [CAHPS] Health Plan Survey	Satisfaction as feasibility component for new intervention	Parents in the intervention group reported higher satisfaction with the referral system and with care overall	Cross-setting: primary care clinic and outpatient clinic; intervention-only: telehealth	
Cook	2010	Satisfaction as a component of the caregiver’s perception of his or her system of care service experiences over the last 6 months	YSS-F	Satisfaction as outcome for established intervention	Higher levels of perceived support related positively to parental satisfaction with services received	Cross-setting: system of care	Also measured child satisfaction
Dvir	2012	Conceptualized parent satisfaction with services as the difference between families’ expectations of care and their actual experience of care. Measured general satisfaction with services and with the evaluation process by psychiatrist/psychiatric nurse clinician, and satisfaction with the follow up and mental health referral process	Study-specific researcher-developed measure	Satisfaction as outcome for established intervention	Most parents were satisfied with services, and reported being better satisfied with service in this program compared to previous contact with mental health providers. No significant group differences in satisfaction by minority status, visit status, or co-morbidity status. High rates of parents reporting they felt prepared, heard, and understood; lower satisfaction was reported with wait times to get services in the community	Primary care	
Farmer	2011	Satisfaction with health services: primary care, specialty care, emergency room, inpatient; and separately, satisfaction with mental health services	Study-specific researcher-developed measure	Satisfaction as outcome for established intervention	Intervention group (care coordination) reported greater satisfaction with mental health services & therapies than the waitlist control group	Primary care (medical home)	Also measured physician satisfaction
Fawley-King	2013	Satisfaction with services for their child: measured overall satisfaction with services, positive outcomes as a result of services, and having support outside the mental health system	YSS-F	Satisfaction in relationship to clinical, population, or service variables	Caregivers were highly satisfied with their child's treatment; higher general satisfaction predicted higher number of caregiver participation activities and the caregiver being more likely to carry out therapist's recommendations at home	Outpatient clinic	
Fawley-King	2014	Mothers' satisfaction with the Triple P program	Study-specific researcher-developed measure	Satisfaction as outcome for established intervention	Mothers reported high rates of satisfaction with the intervention, and almost all reported if they needed help again, they would definitely return to the Triple P program	Outpatient clinic	
Gerdes	2019	Part of engagement & acceptability outcomes	Therapy Attitude Inventory (TAI)	Satisfaction as feasibility component for new intervention	High satisfaction with both arms, but mothers reported higher satisfaction with the culturally-adapted evidence-based treatments (EBTs) than the regular EBT	Cross-setting: outpatient clinic (control) and community-based (intervention)	
Haine-Schlagel	2013	Using parent report to assess perceived effectiveness of treatment	Multidimensional Adolescent Satisfaction Scale (MASS), adapted for parents	Satisfaction in relationship to clinical, population, or service variables	Parents may be more satisfied with care that integrates common elements of evidence-based treatments	Outpatient clinic	
Jacob	2012	3 domains of satisfaction reported to be highly correlated with global satisfaction for pediatric telemedicine patients: 1) technical functioning 2) comfort of patient and provider with the technology and perceived privacy 3) timely and geographic access to care	Parent Satisfaction Survey (Meyers 2008- specifically for telepsychiatry)	Satisfaction as outcome for established intervention	High parental satisfaction	Cross-setting: primary care clinic and telehealth	Also measured primary care physician satisfaction
Kolko	2010	Parent perception of treatment acceptability & helpfulness	CSQ-8	Satisfaction as outcome for established intervention	Intervention group parents (on-site behavioral intervention from pediatric nurse) reported significantly greater satisfaction than control group (enhanced usual care)	Cross-setting: outpatient clinic (control) and primary care (intervention)	
Kolko	2012	Family impressions of services & of the study	CSQ-8	Satisfaction as outcome for established intervention	Intervention group parents (doctor's office collaborative care) were highly satisfied	Primary care	Also measured pediatrician satisfaction
Liddle	2011	Construct to test implementation feasibility	SSS-16	Satisfaction as feasibility component for new intervention	Intervention group children & parents were more satisfied than enhanced SAU group	Cross-setting: juvenile justice detention center, community-based, home-based, and outpatient	Also measured child satisfaction
Mayworm	2020	Measuring three domains of satisfaction important in telepsychiatry: technical functioning, comfort with technology and privacy, and access to care	Parent Satisfaction Survey (Meyers 2008- specifically for telepsychiatry)	Satisfaction as outcome for established intervention	Parents were equally & highly satisfied with telepsychiatry & in-person psychiatry services	Cross-setting: school-based in-person and telehealth	Also measure child & provider satisfaction
Radigan	2014	Measure of appropriateness of care, cultural sensitivity, access, participation, outcomes/functioning, medication management, global satisfaction, and social connectedness	Family Assessment of Care Satisfaction Survey (FACS)- created in New York State; based on national satisfaction survey YSS-F, with local input	Satisfaction as feasibility component for new intervention	Greater proportion of those with access to family peer advocates (FPAs) responded positively (than those without FPAs) to satisfaction overall, and specifically to satisfaction with access to services, appropriateness of services, and participation in services	Cross-setting: inpatient and outpatient	Also measured child satisfaction
Salloum	2015	Acceptability to parents, as a factor affecting implementation	CSQ-8	Satisfaction as outcome for established intervention	Parents & children had high satisfaction levels; parents slightly higher than children	Outpatient clinic	Also measured child satisfaction
Stadnick	2012	Measuring parents’ views on the effectiveness of therapy over the past 5 months	Perceived Effectiveness Subscale of the Multidimensional Adolescent Satisfaction Scale (Caregiver Report Version), plus study-specific researcher developed measure	Satisfaction as outcome for established intervention	Parents reported high level of satisfaction with therapy & that their therapist was effective in working with the child	Outpatient clinic	
Storch	2015	Metric of treatment acceptability	CSQ-8	Satisfaction as outcome for established intervention	Computerized CBT group had high satisfaction rating from both parents & children	Outpatient clinic	Also measured child satisfaction
Thomas	2018	Measuring overall acceptability, effectiveness, and efficiency	Previously validated telemedicine survey (Yip 2003)	Satisfaction as outcome for established intervention	Parents & providers rated high satisfaction; providers slightly lower than parents	Cross-setting: emergency department and urgent care center; intervention-only: telehealth	Also measured provider satisfaction
Trask	2015	Satisfaction as a gauge of quality of care; overall satisfaction with services, positive outcomes as a result of services, and cultural sensitivity scales	YSS-F	Satisfaction in relationship to clinical, population, or service variables	Youth & caregivers had higher satisfaction when clinicians discussed and practiced common elements of evidence-based treatments; consistent across general satisfaction, satisfaction with cultural sensitivity, and satisfaction with outcomes	Outpatient clinic	Also measured child satisfaction
Turchik	2010	Satisfaction as a distinct construct from outcomes; consumer satisfaction important in effectiveness/quality of services, to increase consumer input in service delivery. Parent satisfaction as specifically important because they are involved in treatment plan and bring the child to therapy	The Ohio Scales- Satisfaction Scale	Satisfaction in relationship to clinical, population, or service variables	Parents reported greater satisfaction than the children, but reported lower functioning & higher problem severity scores than children. Parents of younger children reported higher satisfaction; parents of older kids reported lower satisfaction, but the older kids reported higher satisfaction than younger kids. Demographic/clinical outcome variables do not account for much variation. Improvements in functioning/reduction in symptoms were related to satisfaction but small in magnitude	Outpatient clinic	Also measured child satisfaction
Williams	2011	Measure of acceptability	Study-specific researcher-developed measure	Satisfaction as outcome for established intervention	Most parents reported high acceptability scores	Emergency department	Also measured child satisfaction

### Conceptualization

Across the 27 papers that met our criteria for inclusion, the conceptualization of caregiver satisfaction ranged from perspectives of service appropriateness and therapeutic alliance, to ease of access. Four studies [28, 34, 68, 82] aligned with the theoretical model set out by Shafer and Temple [73], by using the YSS-F to measure caregiver satisfaction, defined as a measure of appropriateness of care, cultural sensitivity, access, participation, outcomes/functioning, medication management, global satisfaction, and social connectedness.

Beyond the studies which used the YSS-F, three studies stood out as having well-defined conceptualizations of the measurement of caregiver satisfaction: Acri et al., Dvir et al., and Turchik et al. [3, 32, 83]. Acri et al. [3] defined it as viewing the process and outcomes associated with treatment favorably, and included caregivers’ opinions about helpfulness of groups, the importance of therapy for families, and family improvements as a result of treatment. Dvir et al. [32] described caregiver satisfaction as the difference between families’ *expectations* of care and their actual *experiences* of care. Interestingly, Turchik et al. [83] specifically conceptualized caregiver satisfaction as a distinct construct from outcomes, which makes it worth measuring (i.e., if parent satisfaction correlated perfectly with outcomes, there would be no reason to measure it), and cited caregiver satisfaction as a crucial way to obtain consumer input into service delivery and improve the effectiveness and quality of services. Turchik et al. [83] assert that caregiver satisfaction is specifically important because caregivers are key to both the development of a treatment plan and facilitating their child’s participation in it.

The remaining articles reviewed had less thorough descriptions of their conceptualization of caregiver satisfaction, but a few commonalities among studies emerged. Five studies conceptualized caregiver satisfaction as a measure of engagement or acceptability of a mental health intervention [9, 38, 52, 78, 89, 90]. Three studies explained their measurement of satisfaction as a measurement of caregivers’ perceptions of treatment effectiveness [41, 53, 75]. One study overlapped between several areas, defining satisfaction as treatment acceptability, effectiveness, and efficiency [81]. Two studies conceptualized parent satisfaction as a factor in testing the feasibility of implementation of an intervention [57, 71]. Two telemedicine studies specifically defined satisfaction with relation to technology functioning and comfort, and timely and geographic access to care [47, 59]. Two studies examined treatment satisfaction with different components of multidisciplinary teams that worked with their child [16, 21].

Five studies did not provide definitions or much information about their conceptualization of satisfaction, just simply stating that they measured parent satisfaction with the services the child or family received [19, 25, 27, 33, 35]. Clearly, there are many different definitions and conceptualizations of why and how to measure caregiver satisfaction with their child’s mental health treatment.

### Measures

Satisfaction was measured in a variety of ways. The most common instrument was the CSQ-8, used in 4 of the 27 papers. One other study used the Service Satisfaction Scale- 16 item (SSS-16), which was developed from the CSQ-8. The CSQ was developed in 1979 as a measure of client satisfaction in health and human services systems. It is defined as a standardized measure of general satisfaction with services [55]. The CSQ was originally developed for adults to rate their satisfaction with their own services, but has since been adapted by Tamalpais Matrix Systems, LLC to be used by parents or guardians to “rate the level of quality and effectiveness of services provided to children” [7].

Three studies used the YSS-F, and another used a modification of the YSS-F [68]. The YSS-F was created in 2001 by the Mental Health Statics Improvement Project (MHSIP), a program of SAMHSA, for parents or guardians of youth receiving mental health services. The YSS-F has five subscales: satisfaction, outcomes, access, cultural sensitivity, and participation in treatment. The satisfaction domain is defined as “how well the consumers liked the services overall”, and yet the authors felt that only asking about satisfaction directly was not sufficient. The four additional domains were identified as the elements of mental health services that were most impactful to consumer satisfaction, and were included as part of the questionnaire [70, 73]. Eight of of the 27 studies (30%) used satisfaction measures that were developed specifically for that study, and the remaining studies used a variety of different previously-developed measures, sometimes adapting them to their specific study. In total, 21 different measures of parent satisfaction were used across the 27 studies during the last decade.

### Use

The ways in which caregiver satisfaction data were used in the studies fell into three categories. The first category, satisfaction as a feasibility component for a new intervention, captured 5 of the 27 papers. These articles mostly addressed satisfaction as an added construct for testing the implementation of a new intervention, but not as the main focus of the paper. These papers evaluated the implementation of a new intervention by looking at treatment feasibility or acceptability, which can contain several different elements, one of which is satisfaction. The construct of treatment acceptability, defined as the extent to which consumers (youth, caregivers, and mental health professionals) view the treatment as reasonable, justified, fair, and palatable, has its own history and set of literature. Acceptability may impact family initiation of treatment, engagement, adherence to treatment, and retention in treatments (e.g. a family would be more likely to drop out of a treatment they do not view as acceptable) [50, 54]. Although overlapping in the studies in our review, acceptability is distinct from satisfaction in that it can be measured before treatment has been received, as a general measure of attitudes towards different types of treatment (e.g. [54]. There are tools specifically developed to measure treatment acceptability, such as the Treatment Acceptability Questionnaire, or the Abbreviated Acceptability Rating Profile [54]. However, some researchers may instead use a mix of measures to examine what they may call implementation, acceptability, or feasibility outcomes when investigating a new intervention.

For example, from our review, Gerdes et al. [38] aimed to examine engagement and acceptability outcomes, which they measured as parental attendance, retention, engagement, and satisfaction. Liddle et al. [57] examined implementation outcomes including fidelity, treatment engagement and retention rates, amount of services received, team collaboration measures, and satisfaction with services. However, Radigan et al. [68] focused only on caregiver and youth satisfaction as the central aspect of the paper. They measured parent satisfaction with family peer advocate services (FPAs) they received, and found that those parents who had an assigned family peer advocate responded more positively (than those without FPAs) to satisfaction with services overall, and specifically to satisfaction with access to services, appropriateness of services, and participation in services [68]. This is important, because the development and utilization of family peer advocate programs that assist parents of youth with mental health disorders was in part a response to Unclaimed Children, Revisited (2008) [29].

The second category, satisfaction as an outcome for an established intervention, encompassed 16 of the 27 papers. The testing of caregiver satisfaction with already-developed interventions was used in some papers for testing an intervention in a new setting (e.g. testing telepsychiatry services in different settings) or just as an added measure of effectiveness and acceptability of an intervention.

The third category, satisfaction in relationship to clinical, population, or service variables, included 6 of the 27 papers. Acri et al. [3] used the caregiver satisfaction data measured to assess the relationship between caregiver satisfaction with an intervention (i.e., multiple family group therapy for disruptive behavior disorder) and child and caregiver outcomes. Satisfaction with treatment was predictive of reductions in problematic child behaviors and caregiver stress independently [3]. Turchik et al. [83] measured both parent and child satisfaction with mental health services and investigated the relationship between those satisfactions and youth diagnosis, treatment outcomes, and demographic variables, and found that demographics and clinical outcome variables did not account for much variation. Improvements in functioning and reduction in symptoms were related to satisfaction, but were small in magnitude. Bonach measured various domains of satisfaction in a children’s advocacy center (CAC), and found that satisfaction with services delivered by the CAC (information and logistical coordination, responsiveness and provision of comfort for child victims and non-offending caregivers, and staff courteousness and helpfulness) was important in predicting overall CAC satisfaction [16]. Fawley-King et al. found that higher general satisfaction predicted higher number of caregiver participation activities and the caregiver being more likely to carry out the therapist's recommendations at home [34]. The studies by Haine-Schlagel et al. and Trask et al. both measured caregiver satisfaction as well as evidence-based treatments (EBTs) used by clinicians; parents were more satisfied with care that integrated more common elements of EBTs [41, 82]. This category captured a particular type of analytical approach that helps us to understand the correlates of what drives caregiver satisfaction, but clearly, little of the current research around caregiver satisfaction with their child’s mental health services is focused in this way.

### Results

Table 1 also lists results from the studies included in the review. Because so many different scales were used, it did not seem useful to indicate exact scores on the various measures, but importantly, almost all studies reported high rates of satisfaction, either with the intervention group having greater satisfaction than the control/services as usual group, or with both intervention and comparison group having equally high satisfaction ratings.

### Service setting

The final column in Table 1 lists the setting in which care was delivered that was assessed for caregiver satisfaction. The most common single service setting was an outpatient clinic (10 papers). The second most common single setting was a primary care setting (5 papers). The other two single setting studies were one which measured satisfaction with services in an emergency department, and one which measured satisfaction with services in a children’s advocacy center. The 10 remaining studies assessed caregiver satisfaction with services across multiple settings, including: outpatient clinic, primary care, inpatient, emergency department, juvenile justice detention center, community-based, home-based, telehealth, school-based, urgent care center, and assessing an entire system of care model.

## Discussion

While consumer satisfaction with their general health care services is a well-established construct and clearly linked to engagement in care and lower costs of care [40], caregiver satisfaction with children’s MH services has received less concerted attention. This is surprising, given the fact that all major calls for system reform of the children’s mental health system since Knitzer’s Unclaimed Children [51] have explicitly identified caregiver perspectives and experiences, including satisfaction, as critical to building an effective system for children. Consumer satisfaction may be more well-established than caregiver satisfaction because the passage of the ACA in 2010 mandated the use by Centers for Medicare & Medicaid Services (CMS) of public reporting of consumer satisfaction data, thereby enabling those data to be used to make decisions about hospitals and providers to work with [6]. This valuation of consumer satisfaction may have pushed the field ahead. Additionally, consumer satisfaction may be easier to measure or conceptualize, as it is directly asking an adult about their own care, rather than asking a caregiver about the care their child received, which is less direct and less clearly associated with outcomes.

In contrast, the construct of caregiver satisfaction, and the theoretical models upon which it is based, are the subject of relatively few research articles [(with notable exceptions being [36] and [73] who linked it to other variables of care (e.g. therapeutic alliance, outcomes)]. Our review has shown that there are many differing conceptualizations of how to measure caregiver satisfaction, depending on the service delivery setting and context.

Our review also found that there are no consistently used measures of caregiver satisfaction. The variety of the measures used (21 different measures out of the 27 studies) and the fact that 8 of 27 studies (30%) found no suitable previously developed measure and instead created their own, speaks to the need for refining measurement of caregiver satisfaction.

In addition to the variations in how the construct is measured, our review found that in many of the studies, dropouts are not captured and these studies only assessed satisfaction at the *end* of treatment. Therefore, it is possible that the data were skewed because those who were dissatisfied with treatment may have already dropped out. In addition, the majority of the studies (21 out of 27 or 78%) included satisfaction as an additional measure for testing acceptability or feasibility for new or established intervention, rather than as a primary outcome to examine in relationship to clinical, population, or service variables.

Our review also found that caregiver satisfaction does not have a clear association with improved children’s outcomes. Although there was some predictive power in Acri et al.’s study of satisfaction with treatment predicting reductions in problematic child behaviors and caregiver stress [3], Turchik found find that improvements in children’s functioning and reduction in symptoms were related to caregiver satisfaction, but small in magnitude, that is, the clinical outcome variables did not account for much of the variation [83]. Some studies found satisfaction to be higher in the treatment or intervention group than in the control group (ex: [33, 57, 68]), while others reported equally high satisfaction between both the intervention and control groups (ex: [19, 59]. Even if we were to make the assumption that the intervention groups had better outcomes than control, caregivers may not have been more satisfied with those better outcomes. Interestingly, in almost all of the articles surveyed, the results were described as caregivers being highly satisfied across the board. This begs the question of whether these results are really useful at all in clinical care, or even in determining the feasibility of an intervention for research purposes.

The uniformly high rates of satisfaction may have several different causes. One cause may be that there is a sample bias, in that the interventions studied in research papers may be higher quality than average mental health services provided, so caregivers really are all highly satisfied, or, that only highly satisfied caregivers would choose to fill out a satisfaction survey. Another possibility is that the measurements are not sufficiently sensitive to assess satisfaction levels. Garland et al. [37] posited that satisfaction surveys often have generally positive results and little specificity, lacking clinical utility, such as the CSQ, which has strong internal consistency and reliability, but limited practical utility. Lastly, it is possible that in a system like that of the United States, where at least half of children with mental health problems do not receive needed services [87], that caregivers are satisfied with any services that they can get for their child.

Our review suggests that to be maximally useful, future research on caregiver satisfaction could focus on smaller components that are likely to compose it – caregiver expectancies, perceptions of the benefit of services, and appropriateness of care. A theoretical model of parent engagement from Olin et al. [65] identifies beliefs and expectations, social norms, attitude, and self-efficacy as influences on caregiver engagement. Several studies have identified caregiver expectancies and perceptions, beliefs, and attitudes about care as related to caregiver engagement in their child’s care, and some literature ties those beliefs to treatment outcomes as well [3, 76]. Perceptions of benefit and appropriateness have been shown to *predict* whether families will stick with treatment and continue to receive services; persistence in service-seeking can help drive engagement and treatment completion [44, 91]. It is also interesting to note that these two papers were not captured by our search strategy and thus not included in our review because they never explicitly used the word “satisfaction,” but rather “parental perceptions,” illustrating again the complexity of the caregiver satisfaction construct. However, some other studies more explicitly associated satisfaction with these concepts. Accurso et al. measured caregiver expectancies at baseline, as well as caregiver satisfaction, which they defined as the caregiver’s perception of the effectiveness of services [2]. Multiple studies included in this review mentioned caregiver perception of the effectiveness of services as an element of satisfaction (ex. [41, 75]. Additionally, Dvir et al. conceptualized caregiver satisfaction with services as the difference between families’ expectations of care and their actual experience of care, tapping into the caregiver expectancies element [32].

Other studies examining expectancies, again not included in our review because they did not explicitly used the word “satisfaction”, or were published before 2010, have shown that parent expectancies can affect treatment engagement and retention. For example, one study found that 25.9% of parents noted perception of mental health services as a barrier to retention. The reported barriers under “perception of mental health services” included items such as lacking confidence in the person who recommended they seek help, thinking treatment would not help, not knowing who to trust, and having negative experience with professionals [66]. Another found that negative parent expectancies of outcomes in child psychotherapy predicted lower treatment engagement and premature termination from therapy [64]. Other studies have found that optimistic parent treatment expectations predicted improved therapy outcomes for youths with depression [77] and obsessive–compulsive disorder [56]. These studies as a whole suggest that caregiver satisfaction may be better and more clinically useful if it is assessed in a more nuanced and specific way, by asking about expectancies, benefits, and appropriateness of services throughout the duration of delivery, rather than used as an ad-hoc question at the completion of services. This would allow providers to better identify potential dropouts, make changes to their service delivery, and thus more effectively meet the needs of the family and keep them engaged in care.

Family participation in care was first recognized as an essential element to service quality just over three decades ago [80]. Despite efforts by many States to be responsive to family and youth needs (e.g. family-driven or family-centered care), significant challenges persist. For families to remain the ‘true north’ in the delivery of children’s mental health care services [43] and to ensure that “child mental health is family mental health” [11], our review suggests that efforts be made to not only sharpen the focus of the caregiver satisfaction construct, but to realign it with the quality improvement metrics being developed and deployed across the country as states change their healthcare system models [84]. As these broader transformational healthcare system changes (e.g. alternative payment models) are forcing behavioral healthcare organizations in both the public and private sector toward the provision of value-based care, behavioral health organizations are seeking ways to increase consumer input into their service delivery systems to provide accountability for services [36, 67], making it even more crucial to more assess the components of satisfaction with care with greater accuracy and precision.

## Limitations

While we used a broad range of search terms and crossed many major literatures, the amorphous nature of the construct of caregiver satisfaction with children’s mental health services means that it is possible we missed other studies. The absence of a MeSH heading for caregiver or parent satisfaction with care for their child, difficulty in assessing the measure of satisfaction of care that is not directly derived from the patient under care, and the wide array of measures (and largely developed ad-hoc) being used to assess satisfaction with care, reveal that this is a field still in need of development. Additionally, this review only covered papers which studied the population in the United States, which limits the generalizability of these findings to other countries.

It is important to note that there is currently no commercially available, standardized measure of caregiver satisfaction with their child’s MH care services that has been developed and validated for use in privately delivered care (Olin, personal communication). This is a large gap, as over half of children in the U.S. receive privately delivered care. As value-based care continues to drive and shape the provision of private (and public as well) children’s MH care services, private commercial health care systems will likely press to develop a measure, akin to the adult CAPHS-PC measure of adult patient satisfaction with their behavioral health care services [20].

Our review suggests the need for a revitalized research agenda, which focuses on caregiver expectancies and perceptions of benefits and appropriateness of care, rather than the broader construct of satisfaction. Though not the subject of this paper, research from beyond the U.S. [69], echoes the need to sharpen the construct of caregiver satisfaction with children’s MH services, moving beyond a global notion, and to adapt this construct according to both setting and parent need. Clearly, there is further work needed in this area, and the field could benefit from future studies of the link between caregiver satisfaction and children’s mental health service system reform in other settings, including in other countries. Additionally, a future direction of research that may be useful would be widening the search to compare the construct of caregiver satisfaction in other countries to the construct in the United States. In addition, there is significant potential to integrate the new research approaches being used in the field of user-experience (UX) or human-centered design (HCD) to improve the design and delivery of youth MH services; examples include new evidence-based practice databases designed for *ease of use* by clinicians (e.g., MAP and MATCH; [26] and [86], or digital apps—tailored not just for quick sale but designed according to the needs of parents and children with mental health problems [58]. Using the HCD or user-experience lens to further refine caregiver satisfaction with services might be an important step. The rapid changes in U.S. healthcare are requiring a set of nimble, flexible, precise, and reliable metrics that reflect that important Triple Aim of healthcare reform: consumer experiences of care, improvement in health, and reductions in costs. As health care systems continue to change, including more nuanced assessments of caregiver and youth *experiences of MH care—*both good and bad—will be essential to improving access and quality of services.

## Data Availability

Not applicable.
